# Maternal-Cord Blood Vitamin D Correlations Vary by Maternal Levels

**DOI:** 10.1155/2016/7474192

**Published:** 2016-03-15

**Authors:** Ganesa Wegienka, Hareena Kaur, Roopina Sangha, Andrea E. Cassidy-Bushrow

**Affiliations:** ^1^Department of Public Health Sciences, Henry Ford Hospital, Detroit, MI 48202, USA; ^2^Department of Women's Health, Henry Ford Hospital, Detroit, MI 48202, USA

## Abstract

Vitamin D levels of pregnant women and their neonates tend to be related; however, it is unknown whether there are any subgroups in which they are not related. 25-Hydroxyvitamin D [25(OH)D] was measured in prenatal maternal and child cord blood samples of participants (*n* = 241 pairs) in a birth cohort. Spearman correlations were examined within subgroups defined by prenatal and delivery factors. Cord blood as a percentage of prenatal 25(OH)D level was calculated and characteristics compared between those who did and did not have ≥25% and ≥50% of the maternal level and those who did and did not have a detectable 25(OH)D level. The correlation among Black children was lower than in White children. When the maternal 25(OH)D level was <15 ng/mL, the overall correlation was *r* = 0.16. Most children had a 25(OH)D cord blood level less than half of their mother's; 15.4% had a level that was <25% of their mother's. Winter birth and maternal level were associated with the level being less than 25%. Children with undetectable levels were more likely to be Black and less likely to be firstborn. These data suggest mothers may reduce their contribution to the fetus's 25(OH)D supply once their own level becomes low.

## 1. Introduction

Vitamin D is important for its many health benefits for adults and children. The best evidence for the role of vitamin D in health is related to its importance to bone health [1, 2]. Vitamin D may also be related to other health conditions. For example, early life supplementation in children has been associated with decreased risks of type 1 diabetes and influenza [3, 4]. Despite its positive role in health, vitamin D deficiency is a pandemic [5].

Given the important role of vitamin D in health, there has been much discussion about the best ways to prevent and treat vitamin D deficiency [3, 6]. Prevention in children is a priority as it may relate to skeletal and immune development. Previous research has demonstrated that the vitamin D levels of women and their neonates are highly related [7–11]. The goal of this work was to examine whether there are any particular subgroups in which the mother's prenatal 25-hydroxyvitamin D [25(OH)D] level and her child's cord blood level of 25(OH)D are not strongly related. These analyses could identify pregnant women who need a prenatal vitamin D intervention tailored to them based on the various characteristics, as well as identifying children who may have a lower level of 25(OH)D at birth and may be prioritized for vitamin D screening.

## 2. Methods

### 2.1. Study Population

The birth cohort studied here is part of an NIH and institutionally funded cohort study that enrolled pregnant women receiving care at obstetrics clinics in a health system in the Detroit, Michigan, USA, area for longitudinal study of their children through early childhood with the goal of examining early life exposures related to childhood allergies and asthma. These children and their mothers served as the source population for the analyses. Details of cohort creation have been published [12–14]. Briefly, women were enrolled in their 3rd trimester at which time they provided a blood sample and completed an interview about their health. The child's cord blood was collected at delivery. Only maternal-child pairs in which the child was Black/African American or White/Non-Hispanic/Non-Middle Eastern had their 25(OH)D levels determined as there were insufficient maternal-child pairs for analyses in other race and ethnicity groups. This work was approved by the health system's IRB.

### 2.2. Vitamin D

25(OH)D, representing the sum of 25(OH)D_2_ (ergocalciferol which is diet related) and 25(OH)D_3_ (cholecalciferol which is sun related), was measured in frozen plasma samples (−80°C) in the laboratory of Dr. Neil Binkley at the University of Wisconsin. An HPLC method was used and has been used in previously published research [15–22]. 25(OH)D is expressed in ng/mL. 25(OH)D was measured in the stored samples from pregnancy (3rd trimester) and delivery (cord blood). For those with 25(OH)D levels below the lowest detectable limit of 5 ng/mL, a value of 2.5 ng/mL was assigned. This assignment is a common practice with lab values in research as it allows results to be retained in analyses of continuous measures rather than being removed from analyses due to lack of an actual value.

For the analyses, we examined whether maternal-child correlations in subgroups would vary between those in which maternal 25(OH)D was and was not low. We chose the* a priori* cutpoints of 20 ng/mL and 15 ng/mL. While 20 ng/mL defines deficiency [3], we knew that many of the mothers in the analyses had even lower levels of 25(OH)D. Thus, we also chose to examine the cutpoint of 15 ng/mL. Although limited by sample size, we also examined the associations when maternal levels were less than 40 ng/mL versus levels at least 40 ng/mL (*n* = 218 and *n* = 23, resp.) as it has previously been shown that 40 ng/mL is the level at which 25(OH)D conversion to 1,25-dihydroxyvitamin D_3_ [1,25(OH)_2_D_3_] is maximized [23].

### 2.3. Prenatal Medical Chart Review

The maternal prenatal chart was reviewed for key information used in the analyses including maternal height, parity, delivery type, gestational age at delivery, and the child's birthweight. The maternal weight taken at the first prenatal appointment was recorded and this measurement occurred an average of 7.5 (SD = 5.6) months prior to the child's birth; maternal weight was defined as obese if body mass index (BMI) ≥30 kg/m^2^ or as classes II-III obese if BMI ≥35 kg/m^2^. Child race was based on maternal report in a study-specific interview.

### 2.4. Maternal Atopic Status

Allergen-specific IgE (sIgE) levels in mothers' prenatal blood samples were assessed for a set of allergens (*Dermatophagoides farinae*, dog, cat, timothy grass, ragweed,* Alternaria alternata*, egg, and German cockroach). Maternal atopy was defined as having at least one sIgE ≥0.35 IU/mL.

### 2.5. Statistical Analyses

Descriptive statistics were calculated to provide an overview of the factors and the 25(OH)D levels. We then took a multistep approach to examine the association between the maternal prenatal and cord blood 25(OH)D levels. First, we examined the Spearman correlations for all children and within subgroups. Subgroups were defined by child race (Black or White), delivery type (c-section or vaginal), winter birth (December, January, or February), firstborn status (yes/no), maternal BMI (kg/m^2^ at first prenatal appointment, <30 kg/m^2^/≥30 kg/m^2^, and <35 kg/m^2^/≥35 kg/m^2^), preterm birth (<37 weeks/≥37 weeks), birth weight (≤2500 g/>2500 g), maternal prenatal 25(OH)D, and maternal atopic status (yes/no). These variables were chosen as they are among the most commonly studied characteristics of pregnancy and delivery. Maternal atopic status was included because the primary goal of the cohort was to study allergic outcomes in the children and prenatal and early life vitamin D levels have been investigated for their role in allergic disease development [22, 24–27]. We then calculated cord blood as a percentage of the prenatal level and compared characteristics between those who did and did not have a percentage at least 25% and 50% of the maternal level. Finally, we compared characteristics of those who were and were not above the lowest limit of detection. Factors compared between the groups include child race, winter birth, delivery type, firstborn status, maternal atopic status, birthweight, gestational age, and maternal prenatal level of 25(OH)D.

## 3. Results

There were 241 maternal-child pairs who contributed samples to the analyses. Maternal average age was 30.3 (SD = 5.4) years and most of the children were Black (*n* = 175, 72.6%) and were not born in the winter (81.7%). Almost half the women were obese (46.4%, BMI > 30 kg/m^2^), about a third of the pregnancies were by c-section (36.5%) and most of the children were not firstborn (81.3%).

Descriptive information about the overall prenatal and cord blood levels of 25(OH)D is provided in Table 1. The mean cord blood 25(OH)D level (10.9 ng/mL) is nearly half of the mean maternal prenatal level (23.6 ng/mL). White children and their mothers tended to have higher 25(OH)D levels compared to Black children and their mothers (Tables 1 and 2). Lower prenatal and cord blood levels were found in those who were not firstborn, those mothers with low 25(OH)D levels (<40 ng/mL, <20 ng/mL, and <15 ng/mL), and those who were obese (≥30 kg/m^2^ and ≥35 kg/m^2^). Lower prenatal levels were found among mothers who had a low birthweight infant. No statistically significant differences were found between those who were and were not born in the winter and were of preterm or low birthweight, or by delivery mode or by maternal atopic status.

Spearman correlations between the prenatal-cord blood levels are presented in Table 3. The prenatal and cord blood levels were highly correlated, overall (*r* = 0.75) and for most subgroups including those defined by winter birth, firstborn status, maternal BMI, preterm birth, low birth weight status, delivery type, and maternal atopic status. The correlation among Black children was less than that of the White children, although both correlations were quite strong (*r* = 0.65 and 0.87, resp.). However, there was a notable exception. When maternal levels of 25(OH)D were low, the correlation with the cord blood was weak. For example, when the maternal level was less than 15 ng/mL, the correlation was only 0.16. The correlation was stronger, albeit not as strong as the overall, when the maternal prenatal level was less than 20 ng/mL (*r* = 0.29).

Most of the children had a 25(OH)D cord blood level that was 50% or less of their mother's prenatal level (*n* = 149, 61.8%) and 37 children (15.4%) had a level that was less than 25% of their mother's level. No factors were associated with the level being less than 50% (data not shown); however, there were two factors associated with the level being less than 25%: winter birth and maternal prenatal level. Those children with the percentage less than 25% were more likely to be born in the winter months (29.7% versus 16.2%, *p* = 0.05) and have a lower prenatal maternal level (mean maternal prenatal level = 18.9 ng/mL versus 24.5 ng/mL, *p* = 0.003).

We also examined the characteristics of those children whose 25(OH)D levels were above and below the lowest detectable limit (Table 4). Children who had levels below the detectable limit were more likely to be Black and less likely to be firstborn or have an atopic mother. The mean prenatal level tended to be higher for those who had a detectable 25(OH)D level.

## 4. Discussion

In these analyses of this birth cohort, the correlation between maternal prenatal and cord blood 25(OH)D is quite strong overall. However, the maternal-child correlation is much weaker when the maternal level is low. White maternal-child pairs had higher correlations than the Black maternal-child pairs and Black children had lower percentages of their maternal prenatal 25(OH)D levels. These results are likely due to the generally lower level of 25(OH)D among Black women and suggest that mothers may insufficiently contribute to the child's 25(OH)D supply once their own level falls too low (lower threshold). The data also demonstrate that it is inappropriate to use a prenatal 25(OH)D level to represent the 25(OH)D in a child's early life.

Furthermore, these data suggest that children who are not firstborn will have a lower percentage of their maternal level. This could be attributed to the fact that maternal prenatal 25(OH)D levels were higher in women carrying their firstborn child. This could also reflect that (1) maternal stores may be depleted from prior births and have not recovered and (2) prenatal supplementation in parous women may not be sufficient to eliminate low 25(OH)D levels. In a study of 92 pregnant women in Saudi Arabia, women with two or more previous births were significantly more likely to have lower 25(OH)D_3_ levels compared with those with one previous birth (*p* < 0.05) [28]. The authors also reported a significant correlation between maternal serum and neonatal 25(OH)D_3_ (*r* = 0.89, *p* = 0.01).

Our results complement and, by adding extensive subgroup analyses, add an additional important dimension to previously published analyses of examinations of prenatal-cord correlations and factors predicting cord blood 25(OH)D levels [7, 29]. Nicolaidou et al. reported that neonatal vitamin D had a positive correlation with maternal vitamin D levels (*r* = 0.69, *p* < 0.001) in those mothers with normal vitamin D levels but not in those with hypovitaminosis (*n* = 123 maternal-child pairs) [29]. Godang found a strong positive association between maternal 25(OH)D and cord 25(OH)D (*β* = 0.42, *p* < 0.001) in a subset of 202 Scandinavian women but did not examine the association in subgroups [30]. Bodnar et al. examined whether prepregnancy obesity predicted poor vitamin D status in neonates in their study of 400 women in Pittsburgh, Pennsylvania [7]. Prenatal (4–22 weeks) levels of 25(OH)D were lower for women who were obese before pregnancy compared to women who were lean even after adjusting for season and other factors. This difference likely led to the result in which women who were obese before pregnancy were more likely to have delivered a child with vitamin D deficiency compared to women who had a normal BMI (odds ratio = OR = 2.1, 95% confidence interval = CI 1.2, 3.6). While we did not see differences by antenatal BMI, the work of Bodnar et al. also highlights the importance of examining subgroups.

A limitation of our study is that maternal prenatal 25(OH)D, while measured in the 3rd trimester, was not measured at the same time in the 3rd trimester for all women. We did not have maternal prepregnancy weights and relied on the weight measured at the time of the first prenatal care visit; however, Holland et al. suggest similar BMI categorization based on first measured pregnancy weight and self-reported prepregnancy weight [31]. Furthermore, there may be maternal characteristics that were not collected for these analyses that may identify other subgroups with variable prenatal-cord correlations.

Our goal was to examine whether the maternal-child 25(OH)D correlation varied within subgroups rather than to examine predictors of a child's cord blood 25(OH)D level as previous studies have already highlighted the importance of the maternal level [8, 9, 11, 30]. The results from this birth cohort suggest that a child's 25(OH)D level is only a fraction of their mother's prenatal level. Furthermore, the degree to which the newborn's level is associated with their mother's prenatal level varies by several interrelated factors including maternal race, birth order, and the actual maternal level. Not only do these studies indicate that use of the prenatal level of 25(OH)D to represent the child's early life vitamin D level is not specific, but also the data suggest that a maternal threshold exists below which the mother limits her contribution of 25(OH)D to the fetus. The designs and analytical plans of future studies of prenatal dietary interventions should consider the possibility of a threshold effect when considering the maternal contribution to the fetus. Furthermore, children who are Black and not firstborn and those born to women with very low 25(OH)D may identify a priority group for vitamin D deficiency screening.

## 5. Conclusions

The degree to which a newborn's vitamin D [25(OH)D] level is associated with their mother's prenatal level varies by several interrelated factors including maternal race, birth order, and the actual maternal level. A maternal threshold may exist below which the mother limits her contribution of 25(OH)D to the fetus; low thresholds should be assessed for other prenatal nutrients. The designs and analytical plans of future studies of prenatal dietary interventions should consider the possibility of a threshold effect when considering the maternal contribution to the fetus. Children who are not firstborn, those who are Black, and those born to women with very low 25(OH)D may identify a priority group for vitamin D deficiency screening.

## Figures and Tables

**Table 1:** Descriptive information for 25(OH)D levels in the 241 maternal-child pairs. Values are given in ng/mL^*∗*^.

	Mean	SD	Median	Minimum	Maximum	Number below lowest detectable limit
Prenatal	23.6	11.9	22.7	2.5^*∗*^	64.9	4.98%
Cord blood	10.9	7.4	10.5	2.5^*∗*^	47.5	24.9%
Child is Black (*n* = 175)						
Prenatal	20.1	10.3	19.8	2.5^*∗*^	49.9	6.9%
Cord blood	9.1	6.6	8.5	2.5^*∗*^	47.5	30.2%
Child is White (*n* = 66)						
Prenatal	32.9	10.8	34.1	13.1	64.9	0
Cord blood	15.6	7.2	15.6	2.5^*∗*^	28.7	10.6%

^*∗*^2.5 ng/mL is assigned when the 25(OH)D value is less than the lowest detectable value of 5 ng/mL.

**Table 2:** 25(OH)D levels for prenatal 25(OH)D and cord blood 25(OH)D. Values are in ng/mL.

	*N*	Prenatal		Cord	
		Mean (SD)	Range	Mean (SD)	Range
Baby is Black	175	20.1 (10.3)	2.5–49.9	9.1 (6.6)	2.5–47.5
Baby is White	66	32.9 (10.8)	13.1–64.9	15.6 (7.2)	2.5–28.7

Baby is firstborn	45	27.7 (11.6)	2.5–51.3	13.2 (6.8)	2.5–28.2
Baby is not firstborn	196	22.7 (11.8)	2.5–64.9	10.4 (7.4)	2.5–47.5

Winter birth	44	24.1 (12.2)^*∗*^	2.5–51.3	10.5 (7.4)^*∗*^	2.5–28.2
Nonwinter birth	197	23.5^*∗*^ (11.8)	2.5–64.9	11.0^*∗*^ (7.4)	2.5–47.5

Maternal prenatal 25(OH)D <40 ng/mL	218	21.3 (9.6)	2.5–39.5	9.8 (6.5)	2.5–47.5
Maternal prenatal 25(OH)D ≥40 ng/mL	23	46.1 (6.0)	40.3–64.9	21.9 (6.2)	2.5–28.7

Maternal prenatal 25(OH)D <20 ng/mL	95	12.2 (5.2)	2.5–19.8	6.0 (6.2)	2.5–47.5
Maternal prenatal 25(OH)D ≥20 ng/mL	146	31.0 (8.7)	20.1–64.9	14.1 (6.2)	2.5–28.7

Maternal prenatal 25(OH)D <15 ng/mL	60	9.2 (4.1)	2.5–14.8	5.7 (7.5)	2.5–47.5
Maternal prenatal 25(OH)D ≥15 ng/mL	181	28.4 (9.5)	15.2–64.9	12.6 (6.5)	2.5–28.7

Maternal BMI at 1st prenatal visit <30 kg/m^2^	126	26.8 (12.7)	2.5–64.9	12.1 (7.5)	2.5–35.9
Maternal BMI at 1st prenatal visit ≥30 kg/m^2^	109	20.0 (9.8)	2.5–50.2	9.9 (7.0)	2.5–47.5

Maternal BMI at 1st prenatal visit <35 kg/m^2^	170	25.8 (12.2)	2.5–64.9	11.8 (7.3)	2.5–35.9
Maternal BMI at 1st prenatal visit ≥35 kg/m^2^	65	18.1 (9.1)	2.5–42.2	9.1 (7.3)	2.5–47.5

Preterm birth (<37 weeks)	20	23.1 (14.6)^*∗*^	2.5–51.3	13.8 (9.5)^*∗*^	2.5–35.9
Full term birth (≥37 weeks)	218	23.5 (11.6)^*∗*^	2.5–64.9	10.6 (7.1)^*∗*^	2.5–47.5

Low birth weight (≤2500 g)	17	17.2 (10.5)	2.5–38.9	10.2 (8.1)^*∗*^	2.5–35.9
Not low birth weight (>2500 g)	207	24.0 (12.0)	2.5–64.9	10.9 (7.4)^*∗*^	2.5–47.5

Baby born vaginally	153	24.4 (12.8)^*∗*^	2.5–64.9	11.3 (7.4)^*∗*^	2.5–35.9
Baby born via c-section	88	22.3 (10.0)^*∗*^	2.5–51.3	10.3 (7.3)^*∗*^	2.5–47.5

Mother is atopic	144	22.9 (10.8)^*∗*^	2.5–58.1	11.1 (7.3)^*∗*^	2.5–47.5
Mother is not atopic	90	24.6 (13.6)^*∗*^	2.5–64.9	10.5 (7.6)^*∗*^	2.5–28.7

^*∗*^Indicates *no* statistically significant difference in 25(OH)D levels between those with and without that characteristic at that time point (*p* ≥ 0.05, Wilcoxon Rank Sum test).

**Table 3:** Spearman correlations between prenatal 25(OH)D and cord blood 25(OH)D.

	*r*	*p*
All children	0.75	<0.001

Baby is Black	0.65	<0.001
Baby is White	0.87	<0.001

Baby is firstborn	0.78	<0.001
Baby is not firstborn	0.75	<0.001

Winter birth	0.81	<0.001
Nonwinter birth	0.74	<0.001

Maternal prenatal 25(OH)D <40 ng/mL	0.71	<0.001
Maternal prenatal 25(OH)D ≥40 ng/mL	0.65	<0.001

Maternal prenatal 25(OH)D <20 ng/mL	0.29	0.004
Maternal prenatal 25(OH)D ≥20 ng/mL	0.73	<0.001

Maternal prenatal 25(OH)D <15 ng/mL	0.16	0.22
Maternal prenatal 25(OH)D ≥15 ng/mL	0.77	<0.001

Maternal BMI at 1st prenatal visit <30 kg/m^2^	0.78	<0.001
Maternal BMI at 1st prenatal visit ≥30 kg/m^2^	0.78	<0.001

Maternal BMI at 1st prenatal visit <35 kg/m^2^	0.79	<0.001
Maternal BMI at 1st prenatal visit ≥35 kg/m^2^	0.70	<0.001

Preterm birth (<37 weeks)	0.54	0.015
Full term birth (≥37 weeks)	0.77	<0.001

Low birth weight (≤2500 g)	0.43	0.08
Not low birth weight (>2500 g)	0.77	<0.001

Baby born vaginally	0.78	<0.001
Baby born via c-section	0.71	<0.001

Mother is atopic	0.73	<0.001
Mother is not atopic	0.78	<0.001

**Table 4:** Characteristics of children with 25(OH)D levels above and below the lowest detectable limit.

	Child's level below lowest detectable limit *N* = 60	Child's level *not* below lowest detectable limit *N* = 181	*p*
Baby is Black	88.3%	67.4%	<0.05
Winter birth	23.3%	16.6	0.24
C-section delivery	40%	35.4%	0.52
Child is firstborn	6.7%	22.7%	<0.05
Mother is atopic	50.9%	65.1%	0.05

Birthweight (in grams)	3397 (711)	3370 (608)	0.85
Gestational age (weeks)	38.9 (1.5)	38.7 (2.0)	0.87
Prenatal level (ng/mL)	14.0 (8.7)	26.8 (11.0)	<0.05

Winter is defined as December, January, or February.

*p* for Chi-square (categorical) or Wilcoxon Rank Sum test (continuous).
